# Divergent Evolution among Teleost V1r Receptor Genes

**DOI:** 10.1371/journal.pone.0000379

**Published:** 2007-04-18

**Authors:** Patrick Pfister, Jerome Randall, Juan I. Montoya-Burgos, Ivan Rodriguez

**Affiliations:** 1 Department of Zoology and Animal Biology, University of Geneva, Geneva, Switzerland; 2 National Center of Competence in Research (NCCR) Frontiers in Genetics, University of Geneva, Geneva, Switzerland; Fred Hutchinson Cancer Research Center, United States of America

## Abstract

The survival of vertebrate species is dependent on the ability of individuals to adequately interact with each other, a function often mediated by the olfactory system. Diverse olfactory receptor repertoires are used by this system to recognize chemicals. Among these receptors, the V1rs, encoded by a very large gene family in most mammals, are able to detect pheromones. Teleosts, which also express V1r receptors, possess a very limited V1r repertoire. Here, taking advantage of the possibility to unequivocally identify V1r orthologs in teleosts, we analyzed the olfactory expression and evolutionary constraints of a pair of clustered fish V1r receptor genes, *V1r1* and *V1r2*. Orthologs of the two genes were found in zebrafish, medaka, and threespine stickleback, but a single representative was observed in tetraodontidae species. Analysis of *V1r1* and *V1r2* sequences from 12 different euteleost species indicate different evolutionary rates between the two paralogous genes, leading to a highly conserved *V1r2* gene and a *V1r1* gene under more relaxed selective constraint. Moreover, positively-selected sites were detected in specific branches of the *V1r1* clade. Our results suggest a conserved agonist specificity of the V1R2 receptor between euteleost species, its loss in the tetraodontidae lineage, and the acquisition of different chemosensory characteristics for the V1R1 receptor.

## Introduction

In vertebrates, interindividual interactions related to reproduction are largely dependent on pheromonal communication. Chemosensory structures located in mammals in the nasal cavity and in teleosts in the olfactory rosette represent the major tools used for such exchanges. These structures contain thousands of olfactory sensory neurons, organized in pseudostratified neuroepithelia. Each olfactory sensory neuron extends a single dendrite towards the outside world and an axon which directly connects to the olfactory bulb, in the brain. A single or a few members of a remarkably large olfactory receptor gene repertoire are expressed by each olfactory sensory neuron. Thus, chemical information from the outside world is transported by multiple parallel lines to the olfactory bulb, where it is processed, directed towards various brain areas, and translated into a conscious or unconscious perception.

Four different classes of olfactory receptors have been described in teleosts: odorant, trace amine-associated, vomeronasal type 2 (V2r) and vomeronasal type 1 (V1r) receptors. Odorant receptors number 143 in zebrafish [1], and represent, as in mammals, one of the largest gene families in the genome. Trace amine-associated receptors [2], are also present in fish species (57 genes in zebrafish) [3]. V2rs react to amino acids in teleosts, and are relatively numerous (at least 24 potentially functional V2rs in zebrafish) [4]–[7]. V1rs mediate pheromone detection in mice [8], [9] and are thought to play a similar role in other species. In mammals, V1r genes are highly divergent, often species-specific, and in rodents number over 100 [10]–[14]. The role played by the size and variability of these V1r repertoires is not understood yet. Thus, basic questions, such as the degree of potential redundancy between paralogous V1r genes, remain unanswered. However, positive Darwinian pressure has been shown to act on some of these receptor genes, between paralogous or orthologous sequences [12], [15]–[19], indicating, at least that for some novel genes, novel functions were acquired.

**Figure 1:**
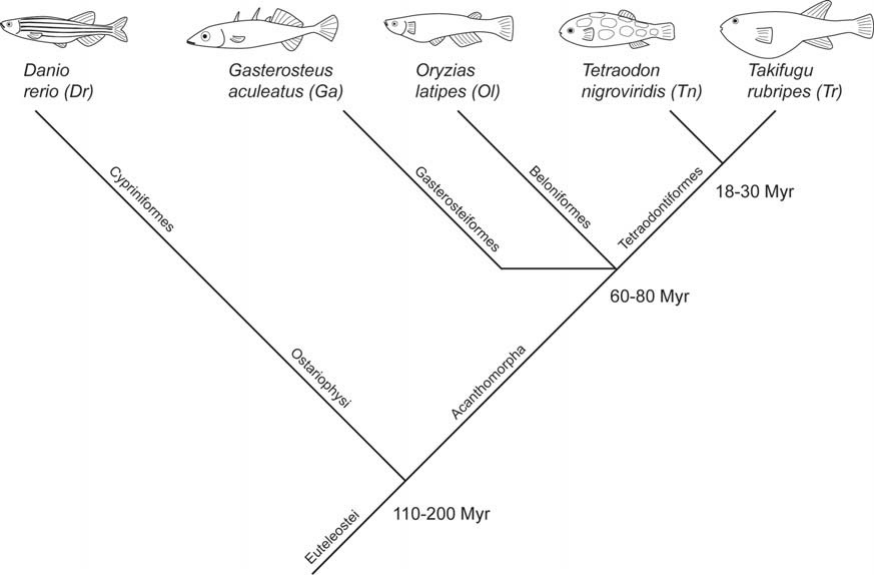
Phylogenetic relationships between zebrafish, stickleback, medaka, tetraodon and fugu. The polytomy at the base of the Acanthomorpha superorder is still debated.

The variability and profusion of V1r genes in some species result from a high rate of gene birth and death[12], [13], [19]. This unusual characteristic renders the identification of orthologous V1r pairs, if existent at the functional level, difficult to perform with certainty, even in species as closely related as mice and rats. Despite the extensive genomic duplications which affected teleost genomes [20]–[24], fish V1r genes appear not to have gone through the flourishing diversification and amplification characterizing mammalian pheromone receptor genes. This peculiar situation allows the study of orthologous V1rs between euteleost species (Table 1) whose radiation dates back over 110 mio. years (Figure 1) [25]–[28]. We here report such an analysis.

**Table 1:** Names, abbreviations, families, orders and superorders corresponding to the teleost species used in this study.

name	abbreviations	family	order	superorder
*Danio albolineatus*	*Da*	Cyprinidae	Cypriniformes	Ostariophysi
*Danio choprae*	*Dc*			
*Danio dangila*	*Dd*			
*Danio kerri*	*Dk*			
*Danio kyathit*	*Dky*			
*Danio malabaricus*	*Dm*			
*Danio nigrofasciatus*	*Dn*			
*Danio pantheri*	*Dp*			
*Danio rerio*	*Dr*			
*Danio yoma*	*Dy*			
*Gasterosteus aculeatus*	*Ga*	Gasterosteoidae	Gasterosteiformes	Acanthomorpha
*Oryzias latipes*	*Ol*	Adrianichthyidae	Beloniformes	Acanthomorpha
*Tetraodon nigriviridis*	*Tn*	Tetraondontidae	Tetraodontiformes	Acanthomorpha
*Takifugu rubripes*	*Tr*			

## Results

### A Linked Pair of Teleost V1r Receptor Genes

A single V1r gene, based on the zebrafish (*Danio rerio, Dr*) (Zv4, 2005), medaka (*Oryzias latipes*) (Medaka 1, 2005), tetraodon *(Tetraodon nigroviridis, Tn)* (Tetraodon 7, 2004) and fugu *(Takifugu rubripes, Tr)* (Fugu 2.0, 2004) assemblies, was previously identified in teleosts [16]. Taking advantage of the newly released zebrafish (Zv6, 2006), medaka (Medaka 1, 2006) and threespine stickleback (*Gasterosteus aculeatus, Ga*) (BROAD S1, 2006) assemblies (see Materials and Methods), and including the fugu (Fugu 4.0, 2005) and tetraodon databases, we performed a search for potential novel V1r sequences. Databases were mined with the TBLASTN and BLASTN algorithms using as queries all known fish V1r, mouse *V1rb2, V1rf3* and *V1re4* sequences. Our criteria for inclusion were an uninterrupted coding sequence of at least 850 nucleotides contained in a single exon (all known vertebrate V1r genes exhibit such characteristics), a potential translated product containing 7 transmembrane segments, and an expected value inferior to 10^−2^.

We identified two different V1r sequences fulfilling these criteria in *Dr*, and found their corresponding orthologs in *Ga* and *Ol*, one of them corresponding to a previously described zebrafish V1r [16]. A single V1r gene was found in *Tn* and *Tr*. We named the V1r common to all tested species *V1r1* and the one missing in the tetraodontidae lineage *V1r2*. We hypothesized the existence of a potential pseudogenized or distant *V1r2* sequence in tetraodontidae species, and tested this hypothesis by searching for sequences with a potential to encode 10 amino acid triplets conserved in *Ol, Dr, and Ga V1r2*: HLA (49), LTR (59), PQT (64), GCK (80), NMA (140), APR (153), GFC (167), TRA (228), FGI (245) and PVV (263), in sequences surrounding tetraodontidae *V1r1* genes (100kb upstream and downstream of tetraodon *V1r1*, and 11kb upstream and 10kb downstream of fugu *V1r1*). We estimated that for a sequence to be considered potentially related to a *V1r2* gene, at least 4 of the 10 conserved triplets were to be present, irrespective of the reading frame or the transcription orientation, and with a distance between each triplet not exceeding+/−12 amino acids relative to the one in the known *V1r2* sequences. No sequence fulfilling these criteria was identified, suggesting the absence of a *V1r2* gene in the vicinity of the *V1r1* tetraodontidae gene (but without excluding the potential presence of a highly degenerate *V1r2* pseudogene).

We also performed PCRs at low stringency and/or used degenerate primers (see Materials and Methods) on genomic *Tr* and *Tn* DNA, using *V1r2* primers located at positions conserved in all other identified fish *V1r2* genes. No amplicon corresponding to a potential *V1r2* gene or pseudogene was obtained, again supporting the potential absence of this gene in Tetraodontidae. However, our own experience makes us wary of such negative observations [16].

**Figure 2:**
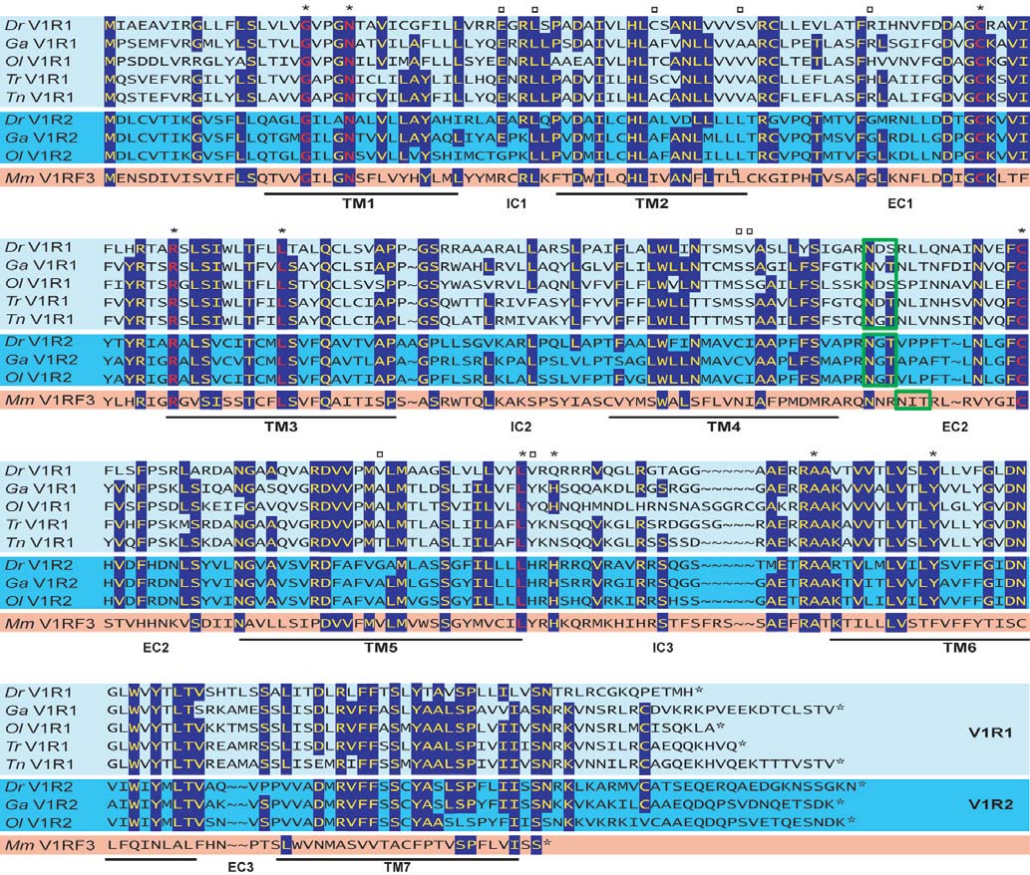
Alignment between teleost V1R1 (light blue) and V1R2 (blue) receptors. *Dr, Ga, Ol, Tr, Tn* and mouse (*Mm*) V1RF3 proteins are shown. Conserved residues (at least seven out of ten) are highlighted in blue. Asterisks indicate conserved residues in virtually all mouse V1Rs, and red letters correspond to the ones also conserved in teleosts. Empty squares show the position of positively selected sites in some genes of the V1r1 clade. Green boxes indicate the position of the conserved N-linked glycosylation sites (NXS/T). TM, transmembrane, IC, intracellular, EC, extracellular domains.

Mammalian V1Rs exhibit 10 highly conserved amino acid residues (some of which are exclusively observed in V1Rs), and a conserved glycosylation site in extracellular loop 2 [14]. 7 of the conserved residues and the glycosylation site were present in all teleost V1r sequences (Figure 2).

To dispose of a larger and therefore more reliable dataset, we then expanded our analysis to species pertaining to a single genus, *Danio*. Using PCR primers specific for *Dr V1r1* and *V1r2* sequences, we investigated the presence of the two genes in the following species: *D. albolineatus (Da), D. choprae (Dc), D. dangila (Dd), D. kerri (Dk), D. kyathit (Dky), D. malabaricus (Dm), D. nigrofasciatus (Dn), D. pantheri (Dp)* and *D. yoma (Dy)* (Table 1).*V1r1* and *V1r2* sequences were found in all tested *Danio* species.

**Figure 3:**
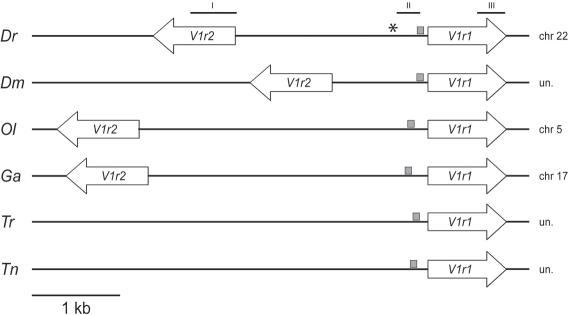
Teleost *V1r1* and *V1r2* genes are clustered in the genome. Relative positions and orientations of *V1r1* and *V1r2* genes in *Dr, Dm, Ol, Ga, Tr* and *Tn. V1r2* was not found in the tetraodontidae species. The grey square represents the position of the conserved *V1r1* 5′UTR region. Horizontal lines above the zebrafish sequence represent the position and size of the RNA probes used for the in situ hybridizations (probes I, II and III) (see Figure 5). The *V1r1* transcription start site is indicated by an asterisk.

Analysis of the genomic location of the two V1r genes indicated that they were clustered on chromosomes 22 in zebrafish, 5 in medaka, and 17 in stickleback. Their transcription directions were inverted in all cases observed, in a bidirectional orientation, with an unusual short distance between coding sequences [14], ranging from 1.1kb (*Dm*) to 3.4 kb (*Ol*) (Figure 3).

### A Conserved Sequence in the 5′UTR of *V1r1*

**Figure 4:**
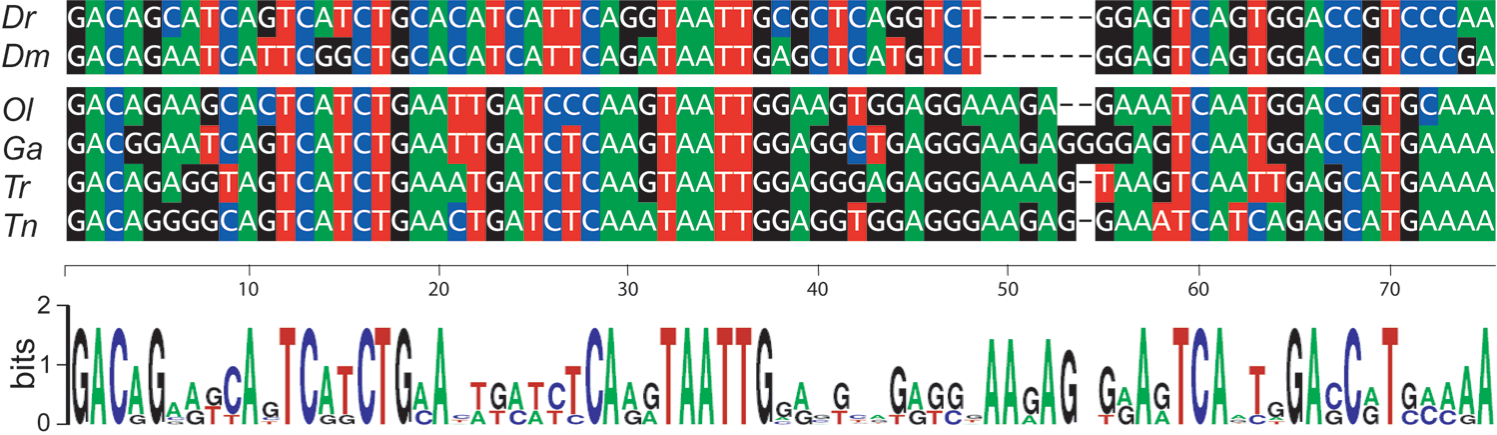
A sequence is conserved in the 5′UTR of *V1r1*. An alignment of the conserved residues in the 5′UTR of *Dr V1r1* mRNA is shown. A 75 base pair region is conserved in all tested species upstream of the translation start site of *V1r1*. A sequence logo was generated.

V1r receptor genes in mice exhibit a characteristic not observed in odorant receptor genes: an unusual conservation of non-coding sequences between members of a given family, which includes transcribed and non-transcribed regions [18]. We evaluated the potential homology of non-coding sequences between orthologous teleost *V1r1* genes (*Dr, Ol, Ga, Tr, Dm*, and *Tn*) by pairwise comparisons of the sequences using the Pipmaker analysis tool. We identified a 75 nucleotide segment located 5′ to the *V1r1* coding sequence containing 31 invariant nucleotides (Figure 4). The position of this conserved sequence relative to the start site ranged from -81 (*Dr*) to -185 (*Ol*) (Figure 3). 5′ RACE was performed and one transription start site was identified 404 bp upstream of the *V1r1* first ATG codon (Figure 3), indicating that the conserved sequence was part of the *V1r1* 5′UTR.

The conserved segment was not found duplicated 5′ to*V1r2* sequence, and was not identified associated with any V1r mouse gene. No significant conservation of other non-coding segments was identified neighboring *V1r2* sequences.

### Expression of *V1r1* and *V1r2* in the Zebrafish Olfactory Rosette

**Figure 5:**
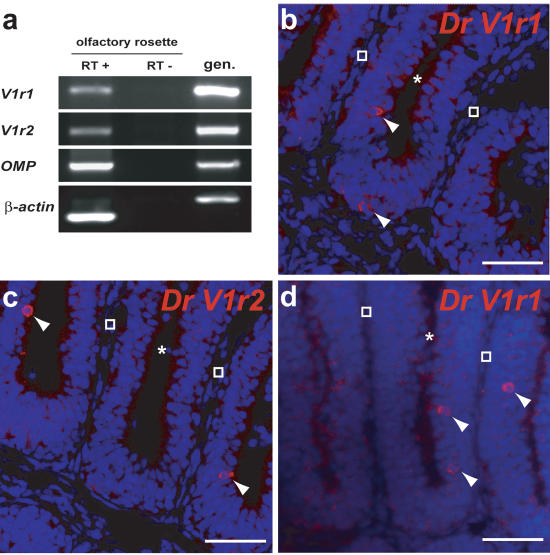
*Dr V1r1* and *V1r2* are expressed in the olfactory rosette. (a) RT-PCR indicating transcription of *V1r1* and *V1r2* in olfactory rosette extracts. OMP (olfactory marker protein) and βactin were used as positive controls. (b) In situ hybridization of a horizontal olfactory rosette section with an anti-sense *Dr V1r1* probe (probe III in Figure 3). (c) In situ hybridization with anti-sense *Dr V1r2* probes (probe I in Figure 3). (d) In situ hybridization with an antisense 5′UTR *V1r1* probe (probe II in Figure 3). Arrows indicate cells reacting to the probes. Asterisks and empty squares correspond respectively to luminal and cartilaginous parts of the rosette. Scalebar: 40 µm.

We investigated the potential expression of *V1r1* and *V1r2* in the olfactory rosette using two approaches. We first performed RT-PCRs on olfactory rosette extracts from male and female adult zebrafish using primers specific for *V1r1* or *V1r2. V1r1* and *V1r2* transcripts were consistently observed in this tissue (Figure 5a). No transcripts were found in other organs, including barbels, lips, heart, gills, muscle and brain (data not shown). Second, and in order to obtain a more precise picture of the cells expressing the V1r receptor genes, we performed in situ hybridizations on olfactory rosette sections. Single neurons located in the sensory neuroepithelium did express *V1r1* transcripts, similarly to what was observed for cells expressing *V1r2* (Figure 5b–d). No evidence of sexual dimorphism in the expression of the V1r receptor genes was observed (data not shown).

### Unequal Evolutionary Rates Between *V1r1* and *V1r2*

**Figure 6:**
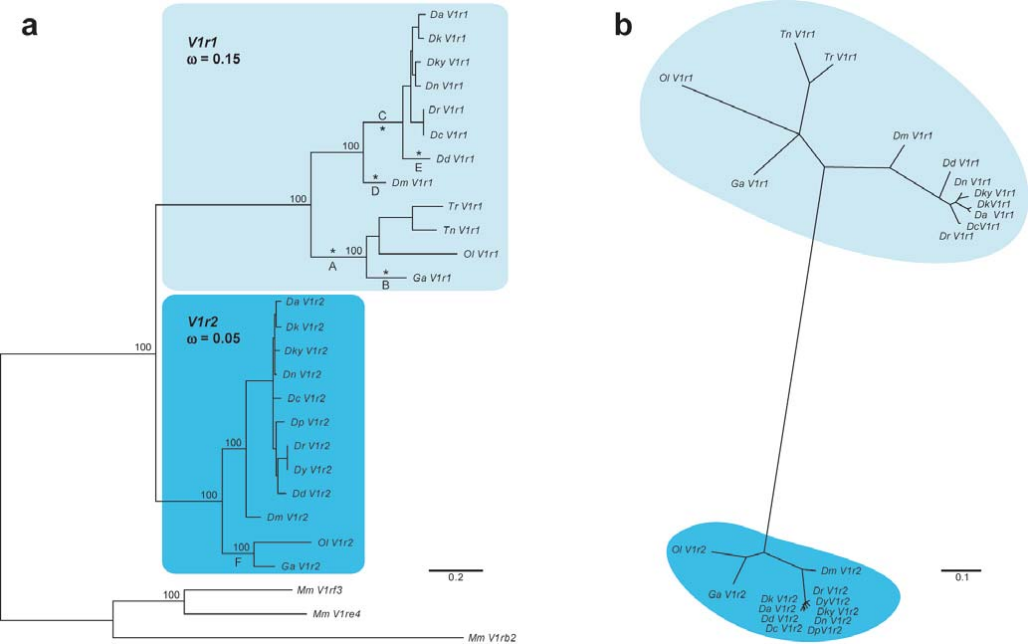
Teleost *V1r1* and *V1r2* exhibit different evolutionary rates. (a) Phylogenetic analysis of teleost V1r genes. A rooted DNA tree was generated, based on an alignment of 24 teleost V1r sequences and three mouse V1rs (*V1rf3, V1re4* and *V1rb2*). Similar ω were obtained for each V1r family using two-or three-ratio models. The V1r1 clade ω value was 3×times higher than the one of the V1r2 clade (see Table 2). Positive selection was detected on branches A–F and asterisks indicate branches in which positively selected sites were identified. (b) Unrooted tree based on the amino acid alignment of the 24 teleost V1r receptors.

Two phylogenetic trees, including 24 *V1r1* and *V1r2* teleost genes or their corresponding proteins, were generated using Maximum Likelihood methods (Figure 6). We evaluated potential evolutionary differences between the V1r1 and V1r2 clades by performing a Relative Rate Test, and found that the V1r1 clade evolved significantly faster than the V1r2 clade (p = 0.015) (see Materials and Methods).

**Table 2:** Likelihood Ratio Tests for substitution models performed on the complete dataset and estimates of the corresponding parameters.

Model	-l	Parameters	LRT
**global models**			
M0 (one-ratio)	6909.549	ω = 0.099	
two-ratio model	6887.725	ωbackground = 0.050, ωV1r1 = 0.147	M0 vs. two-ratio
			2Δl = 43.65, df = 1, p<0.001**
**site-specific models**			
M1a (nearly neutral)	6864.032	p0 = 0.918, (p1 = 0.082	
		ω0 = 0.161	
M2a (positive)	6864.032	p0 = 0.918, p1 = 0.44, (p2 = 0.037)	M1a vs. M2a
		ω0 = 0.086, ω2 = 1	2Δl = 0, df = 2, p = 1
M3 (discrete, K = 2)	6831.373	p0 = 0.555, (p1 = 0.445)	M0 vs. M3
		ω0 = 0.037, ω1 = 0.195	2Δl = 156.35, df = 2, p<0.001**
M3 (discrete, K = 3)	6810.966	p0 = 0.293, p1 = 0.602, (p2 = 0.106)	M3(K = 2) vs. M3(K = 3)
		ω0 = 0.016, ω1 = 0.103, ω2 = 0.455	2Δl = 40.81, df = 2, p<0.001**
M7 (b, neutral)	6822.065	p = 0.823, q = 5.721	
M8 (b, positive)	6817.524	p0 = 0.970, (p1 = 0.030)	M7 vs. M8
		p = 0.942, q = 7.935, ω = 1.001	2Δl = 9.08, df = 2, p = 0,011*
**branch-site models**			
model D (discrete, K = 2)	6813.928	p0 = 0.290, (p1 = 0.710)	M3(K = 2) vs. Model D(K = 2)
		ω0 = 0.256, ω1background = 0.077, ω1V1r2 = 0.014	2Δl = 34.89, df = 2, p = 0.001**
model D (discrete, K = 3)	6806.047	p0 = 0.450, p1 = 0.089, (p2 = 0.461)	M3(K = 3) vs. Model D(K = 3)
		ω0 = 0.131, ω1 = 0.485	2Δl = 9.84, df = 2, p = 0.007*
		ω2background = 0.048, ω2V1r2 = 0.005	Model D(K = 2) vs. Model D(K = 3)
			2Δl = 15.76, df = 2, p<0.001**

-l, likelihood (log) of the tree length

* p<0.05

** p<0.01

To determine the origin of the acceleration detected in the V1r1 clade, we performed a series of tests of increasing sensitivity, looking at the whole dataset or at specific clades, at full-length sequences or codon by codon, and aimed at measuring the selective pressures acting on these genes. Two potential explanations for the observed differential evolutionary rates, which are not mutually exclusive, involve relaxation of the purifying selection, and positive Darwinian selection.

The dN/dS ratio (ω) for the overall dataset was first calculated using the one-ratio model (M0) as implemented in the PAML software (see Materials and Methods). An ω of 0.099 was estimated, suggesting that a strong purifying selection is globally acting on V1r receptors. Using the two-ratio model, the ω estimates for the V1r1 and V1r2 clades were 0.147 and 0.050 respectively (Figure 6). The Likelihood Ratio Test (LRT) indicated that the two-ratio model fit the dataset significantly better than the one-ratio model (Table 2), confirming that the V1r1 clade has a 3×times higher rate of non-synonymous mutations. However, no evidence for positive selection (ω>1) was found.

To further investigate potential changes in selective pressure on specific residues, we applied site-specific models to our dataset (see Materials and Methods). The comparison of different site-specific models suggested heterogeneous evolutionary rates within V1r sequences. Thus, the M3 model (discrete with K = 2) fit the data significantly better than the M0 model (one ratio). The same M3 model allowing 3 different ω, fit the data better than with 2 different ω (Table 2). This last model proposes 29.3% of highly conserved sites (ω0 = 0.016), 60.2% of sites under strong purifying selection (ω1 = 0.103), and 10.6% of the sites under moderate purifying selection (ω2 = 0.455). Furthermore, the M8 model, which allows positive selection and which fit the data better than the M7 model (Table 2), indicated that 3% of the amino acid sites were under positive selection. The identities of the positively selected residues were however not statistically supported (BEB calculations, see Materials and Methods).

We then analyzed our dataset with a site-specific approach, but looking at specific branches. We used the branch-site model D (see Materials and Methods) to identify potential selective pressure differences for a category of sites between the two paralogous clades. The LRTs involving model D (with K = 2 or K = 3 categories of sites) and its nested model M3 indicated that model D with K = 3 fit our data the best (Table 2). This model proposed that for both genes, 45% of sites were under marked purifying selection (ω0 = 0.13), 9% under moderate purifying selection (ω1 = 0.48), and that 46% of the sites displayed a 10×relaxation in the V1r1 clade when compared to the V1r2 clade (ω2V1r2 = 0.005 vs. ω2background = 0.048). However, this large proportion of relaxed sites was still under strong purifying selection (low ω values).

Thus, models with additional categories of ω, applied either to the whole dataset or to specific groups within the tree, were systematically preferred to models of lower complexities (Table 2).

The potential existence of rare positively selected sites in a subset of branches was then assessed. Using again a branch-site model, model A (see Materials and Methods), we analyzed the 21 longest internal and terminal branches of the V1r1/V1r2 clades. We found 5 branches exhibiting positively selected sites in the V1r1 clade and a single branch in the V1r2 clade. Among these branches, we were able to identify 9 significant positively selected sites, 5 of which were located in transmembrane regions (Figure 2). All significant sites pertained to the 5 branches of the V1r1 clade (Table 3, Figure 6).

**Table 3:** Branch-site specific Test 2 for positive selection and branches displaying sites under positive selection (including corresponding positively selected sites).

Branch name	LRT (df = 1)	sites under positive selection^1^	
	Model A vs. Model A with ω2 = 1	95% cutoff	99% cutoff
***V1r1***			
A	2Δl = 5.212, p = 0.0224*	I (145)^2^	-
B	2Δl = 5.619, p = 0.0177*	-	C (144)
C	2Δl = 17.527, p = 0.0000**	L (59)	A (51), H (210)
D	2Δl = 13.123, p = 0.0002**	-	G (71), G (196)
E	2Δl = 9.534, p = 0.0020**	-	E (37), L (40)
***V1r2***			
F	2Δl = 3.916, p = 0.0478*	-	-

1 according to BEB calculations

2 numbers into brackets correspond to the positions indicated in Figure 1

* p<0.05

** p<0.01

In accordance with our previous estimates (results of model M8), the percentage of these residues was limited, ranging from 0.4% to 7.0% (Table 3). 1 to 3 sites per branch displayed an ω significantly above 1 (BEB calculations) (Table 3).

Our approach, involving the use of models of increasing finesse, thus led to the confirmation of differential evolutionary rates between paralogous V1r clades, and to the detection of positive selection between orthologous V1r genes.

## Discussion

We here describe two linked teleost V1r genes, *V1r1* and *V1r2*, and identify their orthologs in multiple fish species, taking advantage of their physical proximity and relative conservation.

Apparently, not all teleosts share the same V1r repertoire; we were indeed unable to identify a *V1r2* receptor gene in the two tested tetraodontidae species. This negative observation may naturally reflect a failure to identify an existing sequence, due to a still incomplete coverage of the tetraodon and fugu genomes. This seems however unlikely since these genomes are close to being fully sequenced. Moreover, since *V1r1* and *V1r2* genes are found in close association in all teleost species tested (less than 3.5 kb apart), and since relatively large genomic sequences surrounding the tetraodon and fugu *V1r1* were available and searched, we propose that our findings reflect a gene loss, which specifically affected the tetraodontidae lineage. This potential genus-specific gene loss is consistent with the remarkably compact genome of members of this lineage, genome that can be as small as 390 Mb in fugu and 340 Mb in tetraodon (while zebrafish and medaka genome sizes are 1.7 Gb and 800 Mb respectively). This limited genomic material translates into a contraction of gene number, shrinking known to affect many gene families, including olfactory receptor gene repertoires. Thus, a comparison between fugu and zebrafish odorant receptor genes indicates a drastic reduction in the fugu repertoire size (143 vs. 44 and 42 odorant receptor genes in zebrafish, fugu and tetraodon respectively) [1].

We identified a short sequence in the 5′UTR of *V1r1* transcripts, conserved between multiple teleost species, including some whose common ancestor dates back over 100 mio. years. This observation is reminiscent of the remarkable 5′UTR conservation present in mouse V1ra and V1rb transcripts [18]. It may point to a transcriptional or translational regulatory element acting on fish *V1r1* genes, and possibly also on *V1r2*. We also report a peculiar genomic organization of the fish *V1r1* and *V1r2* genes. As little as 1.1 kb separates the coding sequences of the two genes, leaving limited space for two 5′UTRs and two promoters since their transcriptional direction is inverted. The zebrafish V1r intergenic sequence, whose very limited size renders it easily amenable to genetic manipulation, will surely prove to be of interest in the study of the mechanisms regulating V1r expression, which at present remain completely unknown. We observed that unequal selective pressures affected the evolution of the V1r1 and V1r2 clades. The V1r1 clade exhibits a relatively relaxed negative pressure, with on given branches, specific residues under positive Darwinian selection, a situation observed for some mammalian V1rs. This finding suggests a possible variable role played by orthologous V1R1 receptors, or at least non-homogenous agonist response profiles between the latter (due for example to species-specific coevolution of agonist-receptor pairs). The situation is clearly different for the V1r2 clade: a strong purifying pressure is at work on V1R2 receptors, suggesting a common role and/or agonist profile between orthologous V1R2 proteins. Such theories will be put to test as soon as we identify natural agonists for fish and mammalian V1rs. But the road is apparently long before we dispose of a general view of agonist-V1r receptor pairs in vertebrates. Indeed, despite an identification of the first V1r genes over a decade ago [29], only a single such pair has been identified [8]. This situation, partly resulting from the difficulties faced to express these receptors in vitro, also reflects a more problematic situation, which is the very limited number of known potential agonists.

## Materials and Methods

### Identification of V1r genes

The following ENSEMBL databases were searched using the BLASTN and TBLASTN algorithms: *Dr* Zv6 (August 2006 release, 7×coverage), *Ga* BROAD S1 (June 2006 release, 11×coverage), *Ol* MEDAKA1 (May 2006 release, 6.7×coverage), *Tn* Tetraodon 7 (September 2004 release, 8.3×coverage), and *Tr* Fugu 4.0 (May 2005 release, 90% coverage). Database mined sequences from *Dr, Ol* and *Ga* were PCR amplified from genomic DNA and sequenced for confirmation. *Da, Dc, Dd, Dk, Dky, Dm, Dn, Dp* and *Dy V1r1 and V1r2* were PCR amplified from genomic DNA using the following primers: TTC CCG GTA ACA CCG CTG TCA TCT G, GAA GAA GAG CCG GAG GTC AGT GAT CAG, TAT GGA CCT GTG TGT CAC, CAG CCT TTA TGA AAC ACA TTC AC, TGG ACC TGT GTG TCA CCA TCA AAA GG, TCA GTT CTT GCC GCT GGA GTT CTT GCC, and sequenced. The *Dm* intergenic sequence was amplified from genomic DNA and sequenced. Conditions for degenerate PCRs performed on fugu and tetraodon genomic DNA were: 4 min. at 95°C followed by 48 cycles of 45 sec. at 95°C, 4 min. at 50°C and 3 min at 72°C, followed by a final extension time of 10 min at 72°C, using the following primers: TCT GSM TCA CCT KCA TKC TGA GYG YST WCC A and GGG CTG AGV GMK GCR TAS MAY GAG GAR AAA AA. Low stringency amplifications were performed with an annealing temperature of 52°C. Sequences were deposited in GenBank with the following accession numbers: DQ887609, DQ887610, DQ887610, DQ887612, DQ887613, AY279523, Q887614, DQ887615, DQ887616, 880989, 884592, 884596, 884602, 884604, 884606, 881043, 881043, 881049, 881053, 884622, and 884628.

### RT-PCR

mRNA was extracted (RNeasy extraction kit, Qiagen) from 40 male and female adult olfactory rosettes. cDNA was synthesized using a random hexamer primer. Primers used for *Dr V1r2*, β-actin and OMP genes were previously described [16]. Primers for *Dr V1r1* were the following: TGT TTC TGT CCC TGG TGC TGG T and AGC CGG AGG TCA GTG ATC AG. PCR conditions were previously described [16].

### 5′ RACE

5′ RACE (Rapid Amplification of cDNA Ends) was performed following the Roche Applied Science RACE kit protocol. First strand cDNA synthesis, and subsequent PCR cycles were performed using the following *Dr V1r1* primers: AGC ACG ATG GCA TCA GCA GGC GAG A, TCG CGG CGC ACC AGC AAG ATG AAG, and CGC AGA TGA CAG CGG TGT TAC C. Amplification conditions were: 3 min. at 95°C, followed by 34 cycles of 15 sec. at 95°C, 30 sec. at 55°C (or 60°C depending on the primer), and 1 min. at 72°C, with a final extension of 10 min. at 72°C.

### 5′UTR Sequence Logo

The 75 base pair sequences corresponding to the *V1r1* 5′UTR homology region of *Dr, Dm, Ol, Ga, Tr*, and *Tn*, initially identified using the Pipmaker analysis tool [30] available at http://pipmaker.bx.psu.edu/pipmaker, were aligned. A logo was generated using the sequence logo software [31] available at http://weblogo.berkeley.edu/logo.cgi.

### In situ hybridizations

*Dr V1r1* and *V1r2* probes corresponded to genomic amplicons obtained with the following primers: ATG TGG TGC CGA TGG TGC TGA TGG, AGC CGT GTG TTG GAG ACG AGG ATC AG, TAT GGA CCT GTG TGT CAC, and TCA TGG AAG TCC ACA TGG CAG AAG. The conserved 5′UTR *V1r1* box probe was flanked by the primers AGT GGA AAT GCA GTG TGC GC and CAA TTA CCT GAA TGA TGT GC. RNA digoxygenin labeled probes were synthesized according to the DIG RNA labeling kit supplier protocol (Roche Molecular Biochemicals). 10 µm cryosections were fixed for 20 minutes with 4% paraformaldehyde at 4°C. Hybridizations were performed in hybridization buffer (50% deionized formamide, 10% dextran sulphate, 1 µg/µl tRNA, 1×Denhardt's, 1×salt solution (2M NaCl, 100mM TRIS, 50mM NaH_2_PO_4_, 50 mM Na_2_HPO_4_, 50mM EDTA, pH 7.5)) overnight at 65°C. Primary anti-DIG antibody (1∶1000) coupled to alkaline phosphatase (Roche Molecular Biochemicals) and FastRed (DAKO) were used for signal revelation. 1 ng/µl DAPI was used to counterstain nuclei. Signal detection was achieved either by an Axiocam camera connected to an Axioplan2 fluorescence microscope (Zeiss), or by confocal imaging using a TCS SP2 AOBS microscope (Leica).

### Phylogenetic trees

Nucleotide sequences of 24 teleost V1r sequences and mouse *V1rb2, V1re4* and *V1rf3* were aligned using ClustalX [32] and manually rearranged using the Bioedit alignment software (v. 7.0.5.3).

The MODELTEST v3.7 program [33] was used to determine the model of DNA sequence evolution that fit our data best. The best fit model was the General Time Reversible (GTR) with a gamma shape distribution of evolutionary rates (α = 1.18; 8 categories of sites). Phylogenetic tree reconstruction based on DNA sequences was performed using the Maximum Likelihood (ML) method as implemented in the program Phyml [34] and using NNI and SPR branch swapping methods, or imposing different starting topologies to avoid local optima. The amino acid tree reconstruction was performed using the JTT model of amino acid changes and a gamma shape distribution of evolutionary rates (α = 2.15; 4 categories of sites). The same topology was retrieved when using two different starting trees: the Bionj Tree or the best topology found using the DNA alignment. For DNA and amino acid analyses, support for branches was assessed by bootstrap analyses of 500 replicates.

### Evolutionary rates and selective pressures

Differences in the evolutionary rates of the two paralogous V1r copies were statistically tested using the RRtree program [35]. Selective pressures acting on the V1r receptor genes were assessed using different models as implemented in the PAML software v.3.15 [36]. Couples of nested models were compared using Likelihood Ratio Tests (LRTs) to statistically determine the best model. Twice the difference of the likelihood values of the tree obtained under each model approximately follows a chi-square distribution and, together with the number of degrees of freedom (df) separating the two models, allows the calculation of the associated *p*-value.

To evaluate a possible unequal ratio of non-synonymous vs. synonymous substitutions (ω) in the two paralogous V1r copies, we performed an LRT comparing the two-ratio model (one ω per clade) with the one-ratio model (M0) where ω is constant. Varying selective pressure along the genes was assessed by a LRT test comparing the site-specific model M3 (discrete) with 2 or 3 classes of sites displaying different ω, against the M0 model (one-ratio). The branch-site model D [37], an extension of the model M3 which allows selective pressure at one class of sites to be different in two parts of the phylogeny, was used to test for divergent selective pressure between the two paralogous V1r genes, by applying an LRT on models D vs. M3.

### Positive selection

Positive selection was first assessed using two LRTs involving site-specific models of increasing complexity. Model M2a (which allows one group of sites to have an ω>1), was compared to model M1a (where ω cannot exceed 1). Discrete model M8 (in which the ω value can vary according to a beta distribution (between 0 and 1) and which includes an extra category of sites with ω>1), was compared to model M7 (in which ω can only vary according to the beta distribution). Branch-site models were used to identify positive selection acting on sites along specific branches of the phylogenetic tree. Model A [38] was used to identify positive selection acting on sites along specific branches of the tree. This model, which allows one category of sites to evolve under positive selection (ω2>1), was compared by LRT to that same model with an ω2 fixed at 1 (Test 2). This LRT test is the most reliable test according to Zhang et al. [38]. Significance of positively selected sites was evaluated using Bayes Empirical Bayes (BEB) calculations [39].

### Animals

Animals were housed and handled accordingly to the guidelines and regulations of the institution and of the state of Geneva.
